# Vascular Aging Estimation Based on Artificial Neural Network Using Photoplethysmogram Waveform Decomposition: Retrospective Cohort Study

**DOI:** 10.2196/33439

**Published:** 2022-03-17

**Authors:** Junyung Park, Hangsik Shin

**Affiliations:** 1 Department of Biomedical Engineering Chonnam National University Yeosu Republic of Korea; 2 Department of Convergence Medicine Asan Medical Center University of Ulsan College of Medicine Seoul Republic of Korea

**Keywords:** Artificial neural network, Cardiovascular risk, Machine learning, Neural network, Photoplethysmogram, Vascular aging

## Abstract

**Background:**

For the noninvasive assessment of arterial stiffness, a well-known indicator of arterial aging, various features based on the photoplethysmogram and regression methods have been proposed. However, whether because of the existing characteristics not accurately reflecting the characteristics of the incident and reflected waveforms of the photoplethysmogram or because of the lack of expressive power of the regression model, a reliable arterial stiffness assessment technique based on a single photoplethysmogram has not yet been proposed.

**Objective:**

The purpose of this study is to discover highly correlated features from the incident and reflected waves decomposed from a photoplethysmogram waveform and to develop an artificial neural network-based regression model for the assessment of vascular aging using newly derived features.

**Methods:**

We obtained photoplethysmograms from 757 participants. All recorded photoplethysmograms were segmented for each beat, and each waveform was decomposed into incident and reflected waves by the Gaussian mixture model. The 26 basic features and 52 combined features were defined from the morphological characteristics of the incident and reflected waves. The regression model of the artificial neural network was developed using the defined features.

**Results:**

In correlation analysis, the features from the amplitude of the reflected wave and the skewness of the photoplethysmogram showed a relatively strong correlation with the participant’s real age. In the estimation of real age, the artificial neural network model showed 10.0 years of root mean square error. Its estimated age and real age had a strong correlation of 0.63 (*P*<.001).

**Conclusions:**

This study proved that the features defined from the reflected wave and skewness of the photoplethysmogram are useful to assess vascular aging. Moreover, the regression model of artificial neural network using these features shows the feasibility for the estimation of vascular aging.

## Introduction

Arterial stiffness is one of the major factors to clinically assess the risk of cardiovascular disease [1]. Hemodynamically, it is known that arterial stiffness increases with aging because of the change of arterial composition and the reduction of arterial elasticity [2]. Therefore, it is possible to objectively grasp the aging status of arteries through arterial stiffness. An increase in arterial stiffness indicates the aging of blood vessels, while a decrease in arterial stiffness indicates the health of blood vessels [3,4]. In previous studies, the assessment of arterial stiffness was conducted with features or blood pressure values extracted from continuous blood pressure waveforms [5-9]. Murgo et al [5] observed continuous changes in arterial stiffness with age using the augmentation index (AIx), which is defined as the percentage of central augmented pressure to central pulse pressure of the blood pressure waveform. According to a study by McEniery et al [7], it is possible to accurately measure arterial stiffness using the AIx calculated from aortic blood pressure waveforms, but it is reported that AIx is a sensitive marker only for those under 50 years of age. In the assessment of arterial stiffness with AIx, continuous blood pressure waveforms must be measured in an invasive way. Thus, it puts a burden on the patients and is difficult to measure in daily life. Antza et al [9] classified the presence or absence of early vascular aging from the blood pressure data using the machine learning method of random forest. However, Antza et al [9] only determined the presence or absence of vascular aging but could not explain the continuous process of vascular aging.

Photoplethysmogram (PPG), which is a noninvasive optical measuring technique of blood volume changes in microvessels, was also used to assess arterial stiffness. In the PPG waveform, the systolic phase and the diastolic phase repeatedly appear, corresponding to the cardiac systole and the cardiac diastole. The systolic phase indicates an increase in vascular blood volume, and the diastolic phase indicates a decrease in vascular blood volume [10]. Millasseau et al [11,12]addressed that the PPG waveform is formed by the superposition of the incident wave and the reflected wave of the blood pressure. The incident wave is generated by cardiac systole, and the reflected wave is generated by impedance mismatch at arterial bifurcation points. Dawber et al [13]also analyzed how the shape of PPG waveform changes according to the increase in arterial stiffness due to aging. They found that as aging progresses, the diacritic notch of the PPG waveform gradually disappears and the returning time of the reflected wave is shortened. Therefore, their study showed that changes of the PPG waveform could be used to evaluate arterial stiffness. Millasseau et al [14,15] derived the stiffness index (SI) based on the time difference between the systolic and diastolic peaks of PPG, and reported that SI has a significant difference according to vascular aging. Further, Yousef et al [16] calculated the reflection index (RI) as the ratio of PPG’s systolic amplitude to diastolic amplitude and showed that RI significantly increased with age. However, the RI and SI introduced in both studies are obtained from the summed waveform of the incident and reflected waves of the PPG, despite the concept being derived from the individual incident and reflected waves of the PPG. Therefore, these features cannot be said to accurately reflect the incident and reflected wave characteristics of the PPG and may be influenced by other external factors. Park et al [17] used the wave decomposition method to define features and develop the vascular assessment model in their study. They decomposed a PPG waveform into an incident wave and a reflected wave and defined features from the waves, directly reflecting the incident and reflected characteristics of PPG. They then confirmed that the defined features had a higher correlation with age than RI and SI and developed a regression model for vascular aging assessment.

In recent studies, machine learning techniques have been introduced to evaluate arterial stiffness. Dall’Olio et al [18] created a convolutional neural network (CNN)-based vascular aging assessment model, which used the PPG raw signal measured by smartphone as an input. Their CNN-based model showed similar performance to the existing PPG feature-based model, and it verified that the machine learning models have the possibility of vascular aging assessment with input data measured from a wearable device. Chiarelli et al [19] estimated the actual age of participants from PPG and electrocardiogram (ECG) measurement, using a deep convolutional neural network (DCNN) model. Their DCNN model showed the result of 7-year-old root mean squared error (RMSE), which has a higher performance in vascular aging estimation than the PPG-feature-based multiple regression and artificial neural network (ANN) models.

The purpose of this study is to develop a new vascular aging assessment model using the PPG, which could be noninvasively and easily measured in daily life. In particular, unlike the existing PPG-based vascular aging estimation studies, we decompose the incident and reflected waves of the PPG waveform. New highly correlated features are then explored for vascular aging assessment from the decomposed PPG waves. Lastly, an ANN-based regression model with excellent nonlinear estimation performance is applied to estimate vascular aging.

## Methods

### Data and Ethical Considerations

Data were obtained from a total of 1000 patients who were scheduled for elective surgery (thyroid, breast, or abdominal) from July to September 2015 at Asan Medical Center. Through cross-checking of two researchers, 17 participants with loss of signal and 226 participants with indistinguishable PPG waveforms were excluded from the analysis. As a result, data from a total of 757 participants were used. Table 1 shows the summarized characteristics of 757 participants included in the analysis. The PPG waveform was obtained using a pulse oximeter (E^2^-KIT; KTMED, Co Ltd), and the PPG Probe was placed between the nasal column and the nasal septum as a transmit type. Signals were recorded at 125 or 250 Hz sampling frequency for 5 min. Data acquisition was performed after obtaining approval from the Asan Medical Center (Songpa-gu, Seoul, South Korea) Research Ethics Committee (IRB No.2015-0104).

**Table 1:** Characteristics of patients included in the analysis (N=757).

Category		Values
**Sex, n (%)**		
	Male	348 (46.0)
	Female	409 (54.0)
**ASA PS^a^, n (%)**		
	PS 1	450 (59.4)
	PS 2	277 (36.6)
	PS 3	30 (4.0)
Weight (kg), median (range)		61.8 (54.1-69.4)
Height (cm), median (range)		161.6 (155.7-168.0)
BMI (kg/m^2^), median (range)		23.5 (21.3-25.9)
**Age (years)^b^, n (%)**		
	0-29	10 (1.3)
	30-39	61 (8.1)
	40-49	168 (22.2)
	50-59	215 (28.4)
	60-69	177 (23.4)
	70-79	108 (14.3)
	80-89	18 (2.4)
**Social characteristics**		
	Smoking	111 (14.7)
	Alcohol	240 (31.7)
**Medical history (multiple answers possible)** **, n (%)**		
	Hypertension	213 (28.1)
	Diabetes mellitus	90 (11.9)
	Pulmonary disease^c^	15 (2.0)
	Renal disease^d^	5 (0.7)
	Hepatic disease^e^	23 (3.0)
	Neurologic disease^f^	8 (1.1)
	Others^g^	16 (2.1)

^a^ASA PS: American Society of Anesthesiologists Physical Status((1) a normal healthy patient, (2) a patient with mild systemic disease, and (3) a patient with severe systemic disease).

^b^The median age is 56 years, with a range of 46-65 years.

^c^Pulmonary disease: asthma (7), emphysema (1), bronchiectasis (1), chronic obstructive pulmonary disease (5), and old tuberculosis (1).

^d^Renal disease: chronic kidney disease (2) and end stage renal disease (3).

^e^Hepatic disease: hepatitis B virus (11), hepatitis C virus (2), and liver cirrhosis (10).

^f^Neurologic disease: stroke (1) and cardiovascular accident (7).

^g^Others: angina (12), carotid artery stenosis (1), iron deficiency anemia (1), hyponatremia (1), and intracranial hemorrhage (1).

### Preprocessing

**Figure 1:**
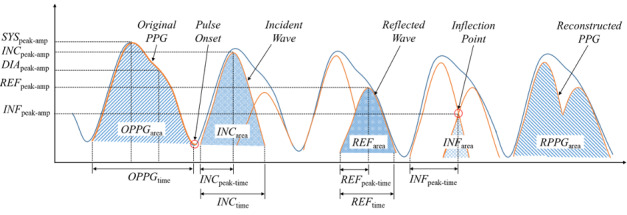
Characteristics of the original PPG, incident and reflected waves, and reconstructed PPG for deriving candidate features. DIA: diastolic; INC: incident wave; INF: inflection point; OPPG: original photoplethysmogram; PPG: photoplethysmogram; REF: reflected wave; RPPG: reconstructed photoplethysmogram; SYS: systolic.

The measured signal was filtered using a finite impulse response bandpass filter having a 0.5-10 Hz passband, and then the pulse onset (ie, the start point of the waveform for each pulse) was detected (Figure 1). Based on the detected pulse onset, each participant’s PPG was divided into pulses to generate segments. At that time, an arrhythmic waveform with an irregular PPG interval or amplitude, or an abnormal waveform with a maximum diastolic amplitude (*DIA*_peak-amp_) greater than the systolic maximum amplitude (*SYS*_peak-amp_), was excluded from the analysis. Since the number of samples for each segment was different due to the nonuniform heartbeat interval for each participant, linear interpolation was performed so that each segment had the same number of samples (ie, 1000). Since the PPG amplitude measured from each participant has an arbitrary value, it was converted to the value between 0 and 1 using the min-max normalization method.

### Features

The features for vascular aging assessment consist of a basic feature defined from the specific points of the waveform before and after the decomposition of the incident and reflected waves of the PPG and a combined feature generated by combining the basic feature. Gaussian mixture model [20] was used for PPG waveform decomposition. Figure 1 shows that through waveform decomposition, each PPG segment was divided into two partial waveforms, one incident wave, and one reflected wave. The evaluation of the appropriateness of the PPG waveform decomposition was performed by calculating the correlation coefficient between the reconstructed PPG and the original PPG and comparing the decomposed waveform feature points. In the verification process, only those segments in which the correlation coefficient between the PPG waveform reconstructed from the decomposed incident and reflected waves and the original PPG was 0.9 or more, and the amplitude of the peak of the incident wave (*INC*_peak-amp_) was greater than the amplitude of the peak of the reflected wave (*REF*_peak-amp_), were used for analysis.

From the waveforms before and after the decomposition of the incident and reflected waves of the PPG, 26 basic features were generated for the development of the vascular aging estimation model. Table 2 shows the features that are defined as follows: 12 features from the amplitude and time of the maximum peak, and total area, total time, skewness, and kurtosis in each waveform of the incident and reflected waves; 3 features from the amplitude, time, and area under the inflection point where the incident wave and the reflected wave intersect; and 3 features from the area, skewness, and kurtosis of the PPG reconstructed by combining the incident and reflected waves. In addition, 8 features were defined from the feature points indicating the amplitude and time of the systolic and diastolic peaks, the total area and time, and the skewness and kurtosis of the original PPG before the decomposition of the incident and reflected waves. Textbox 1 shows 52 combined features, which were defined as ratios or differences of the 26 basic features after dividing them into time-related features and amplitude-related features. A total of 78 candidate features were generated to develop a regression model for ANN-based vascular aging estimation. All preprocessing and feature extraction processes were performed using Matlab 2018b (Mathworks).

**Table 2:** Basic features defined from incident and reflected waves, first inflection point, reconstructed PPG^a^, and original PPG.

Pulse type and feature		Definition
**Incident wave**		
	*INC* ^b^_peak-amp_	Amplitude of incident wave’s peak
	*INC*_area_	Area of incident wave
	*INC*_peak-time_	Time of incident wave’s peak
	*INC*_time_	Time period of incident wave
	*INC*_skew_	Skewness of incident wave
	*INC*_kurt_	Kurtosis of incident wave
**Reflected wave**		
	*REF* ^c^_peak-amp_	Amplitude of reflected wave’s peak
	*REF*_area_	Area of reflected wave
	*REF*_peak-time_	Time of reflected wave’s peak
	*REF*_time_	Time period of reflected wave
	*REF*_skew_	Skewness of reflected wave
	*REF*_kurt_	Kurtosis of reflected wave
**First inflection point**		
	*INF* ^d^_peak-amp_	Amplitude of first inflection point
	*INF*_peak-time_	Time of first inflection point
	*INF*_area_	Area of first inflection
**Reconstructed PPG**		
	*RPPG* ^e^_area_	Area of reconstructed PPG
	*RPPG*_skew_	Skewness of reconstructed PPG
	*RPPG*_kurt_	Kurtosis of reconstructed PPG
**Original PPG**		
	*SYS* ^f^_peak-amp_	Amplitude of systolic peak
	*SYS*_peak-time_	Time of systolic peak
	*DIA* ^g^_peak-amp_	Amplitude of diastolic peak
	*DIA*_peak-time_	Time of diastolic peak
	*OPPG* ^h^_area_	Area of original PPG
	*OPPG*_time_	Time period of original PPG
	*OPPG*_skew_	Skewness of original PPG
	*OPPG*_kurt_	Kurtosis of original PPG

^a^PPG: photoplethysmogram.

^b^INC: incident wave.

^c^REF: reflected wave.

^d^INF: inflection point.

^e^RPPG: reconstructed photoplethysmogram.

^f^SYS: systolic.

^g^DIA: diastolic.

^h^OPPG: original photoplethysmogram.

**Combined features derived from the basic features in the spatial, temporal, and spatiotemporal domains. INC: incident wave; REF: reflected wave; RPPG: reconstructed photoplethysmogram; SYS: systolic; OPPG: original photoplethysmogram.**

Domain and feature

**Spatial**

- *INC*^a^_peak-amp_/*INC*_area_
- *INC*_peak-amp_/*REF*^b^_peak-amp_
- *INC*_peak-amp_/*REF*_area_
- *INC*_peak-amp_/*RPPG*^c^_area_
- *INC*_area_/*REF*_peak-amp_
- *INC*_area_/*REF*_area_
- *INC*_area_/*RPPG*_area_
- *REF*_peak-amp_/*REF*_area_
- *REF*_peak-amp_/*RPPG*_area_
- *REF*_area_/*RPPG*_area_
- *INC*_peak-amp_–*REF*_peak-amp_
- *INC*_area_–*REF*_area_
- *SYS*^d^_peak-amp_–*INC*_peak-amp_
- *SYS*_peak-amp_–*REF*_peak-amp_

**Temporal**

- *INC*_peak-time_/*INC*_time_
- *INC*_peak-time_/*REF*_peak-time_
- *INC*_peak-time_/*REF*_time_
- *INC*_peak-time_/*OPPG*^e^_time_
- *INC*_time_/*REF*_peak-time_
- *INC*_time_/*REF*_time_
- *INC*_time_/*OPPG*_time_
- *REF*_peak-time_/*REF*_time_
- *REF*_peak-time_/*OPPG*_time_
- *REF*_time_/*OPPG*_time_
- *REF*_peak-time_–*INC*_peak-time_
- *OPPG*_time_–*INC*_peak-time_
- *OPPG*_time_–*REF*_peak-time_

**Spatiotemporal**

- *INC*_peak-amp_/*INC*_peak-time_
- *INC*_peak-amp_/*INC*_time_
- *INC*_peak-amp_/*REF*_peak-time_
- *INC*_peak-amp_/*REF*_time_
- *INC*_peak-amp_/*OPPG*_time_
- *INC*_area_/*INC*_peak-time_
- *INC*_area_/*INC*_time_
- *INC*_area_/*REF*_peak-time_
- *INC*_area_/*REF*_time_
- *INC*_area_/*OPPG*_time_
- *REF*_peak-amp_/*INC*_peak-time_
- *REF*_peak-amp_/*INC*_time_
- *REF*_peak-amp_/*REF*_peak-time_
- *REF*_peak-amp_/*REF*_time_
- *REF*_peak-amp_/*OPPG*_time_
- *REF*_area_/*INC*_peak-time_
- *REF*_area_/*INC*_time_
- *REF*_area_/*REF*_peak-time_
- *REF*_area_/*REF*_time_
- *REF*_area_/*OPPG*_time_
- *RPPG*_area_/*INC*_peak-time_
- *RPPG*_area_/*INC*_time_
- *RPPG*_area_/*REF*_peak-time_
- *RPPG*_area_/*REF*_time_
- *RPPG*_area_/*OPPG*_time_

### Artificial Neural Network Regression Model

**Figure 2:**
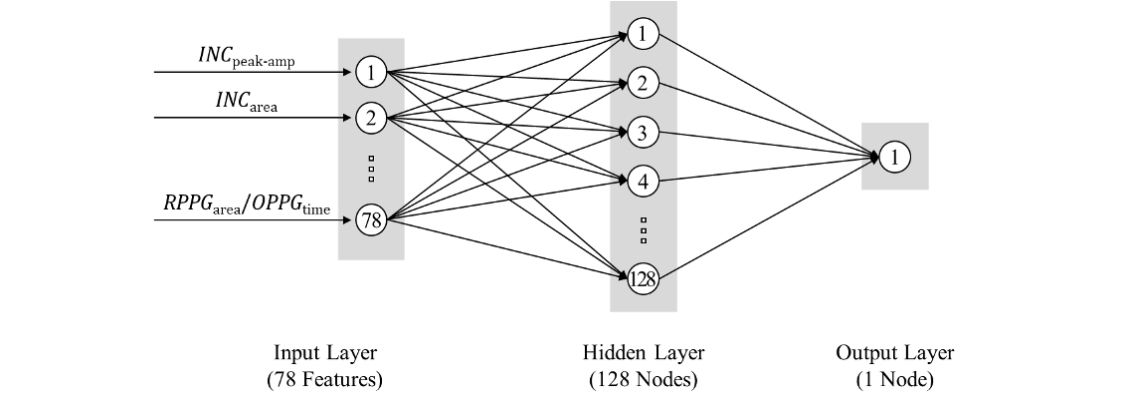
Architecture of the optimal version of the ANN-based regression model developed in this study. ANN: artificial neural network; INC: incident wave; OPPG: original photoplethysmogram; RPPG: reconstructed photoplethysmogram.

In this study, since the actual age of participants is estimated based on various features extracted from their PPG, we used the ANN model, which is frequently used for nonlinear regression with independent features. Table 3 shows that an ANN-based regression model for estimating vascular aging was developed and evaluated using the parameters of various conditions. As a result, the model showing the optimal performance was found as indicated in bold. Figure 2 shows that the developed ANN-based regression model consists of an input layer, a hidden layer, and an output layer. The input layer consists of 78 nodes that receive the features defined by the PPG as inputs. The hidden layer consists of a single layer with 128 nodes. The output layer consists of a single node that outputs the age of the participants estimated through calculation in the hidden layer. Rectified linear unit was used as the activation function [21]. Dropout, which removes hidden layer nodes at a certain rate, was applied with the dropout rate of 0.5. Adam optimizer and learning rate of 0.001 were applied to train the model.

A leave-one-out cross-validation (LOOCV) was used for the development and testing of the ANN-based regression model. In LOOCV, the entire data was divided into one test set and the rest assigned to the model development set. The model development set was divided into a training set and a validation set at a ratio of 8:2 with the same age distribution of participants. After training the model with the development set, the model was evaluated with the test set, and this process was repeated as many times as the number of data, so that all data were used for the model evaluation. The final performance of the model was obtained by averaging each evaluation result. The regression performance of the developed model was represented as RMSE. The ANN-based regression model proposed in this study was developed using 2.90 GHz Intel Core i7-10700 processor, 64 GB 1,333 MHz DDR4 RAM, NVIDIA Geforce RTX 2070 Super, Python 3.6.7: Anaconda, and Tensorflow 2.3.0.

**Table 3:** Different values of hyperparameters for ANN^a^-based regression model for the estimation of vascular aging. Bold type indicates the hyperparameters for the optimal model.

Parameter	Value
Input Layer Nodes	**78**
Output Layer Nodes	**1**
Hidden Layers Number	**1** 2 3 4
Hidden Layer Nodes	64 **128** 256 512 1024
Activation Function	**ReLU^b^**
Dropout Probability	0 0.1 0.3 **0.5**
Kernel Initializer	**He_uniform**
Loss Function	**MAE^c^**
Learning Rate	0.01 0.005 **0.001** 0.0005 0.0001
Optimizer	SGD^d^ **Adam**
Early Stopping Patience	30 **50**
Input Data Scaler	Standard **Robust**

^a^ANN: artificial neural network.

^b^ReLU: rectified linear unit.

^c^MAE: mean absolute error.

^d^SGD: stochastic gradient descent.

### Statistical Analysis

The Pearson correlation coefficient was calculated to investigate the relationship between the participants’ actual age and each feature, which was defined from the waveforms before and after the decomposition of PPG into the incident and reflected wave. The RMSE and coefficient of determination of the age estimated by the ANN-based vascular aging estimation model, which was developed with all the PPG features defined in this study, were calculated. In addition, using the estimated age and the actual age, a scatter plot and a Bland-Altman plot were made and used to analyze the model's estimation performance.

## Results

### Correlation Analysis

The results of the correlation analysis between the actual age and the PPG features are as follows. The correlation coefficient between the actual age and the basic features, which is defined from the original PPG, the incident and reflected waves decomposed from PPG, and the reconstructed PPG, is shown in Table 4. The reflected wave and the reconstructed PPG-related features showed a high correlation with the actual age. Among the reflected wave and reconstructed PPG features, *REF*_peak-amp_, *REF*_area_, *RPPG*_area_, and *RPPG*_skew_ showed a correlation greater than a weak correlation (|R|>0.3), and their correlation coefficients were –0.42, –0.45, and –0.45, respectively. However, most of the features defined from the incident wave and the first inflection point showed a very weak correlation or did not have significant correlation with age. Among the features defined from the original PPG signal, the features using the diastolic peak or skewness feature of the PPG waveform showed a high correlation with age. The features showing the highest correlation in each type of pulse were *SYS*_peak-time_, *DIA*_peak-amp_, and *OPPG*_skew_, and their correlation coefficients were 0.27, –0.39, and 0.41, respectively. Individual features showed a high correlation with age in the order of *REF*_area_, *REF*_peak-amp_, and *OPPG*_skew_, and their correlation coefficient values ​​were –0.45, –0.42, and 0.41, respectively. However, *INC*_peak-amp_, *REF*_time_, *INF*_area_, *RPPG*_kurt_, *SYS*_peak-amp_, *OPPG*_area_, and *OPPG*_kurt_ showed no statistically significant correlation with the actual age, and their *P* values were .10, .51, .28, .23, .52, .12, and .05, respectively. Table 5 shows the correlation between the actual age and the combined features created by the combination of the basic features. In comparing the amplitude domain feature and the temporal domain feature, some features in the spatial domain feature showed more than a weak correlation (|R|>0.3) with the actual age, but most of the temporal domain feature showed no correlation or only a very weak correlation (|R|<0.3) with the actual age. As a result, it was found that the combined feature in the spatial domain has a higher correlation with age than the combined feature in the temporal domain. Among the spatial domain features, *INC*_area_/*REF*_peak-amp_, *INC*_area_/*REF*_area_, and *SYS*_peak-amp_ –*REF*_peak-amp_ showed high correlation with age, and their correlation coefficients were 0.38, 0.37, and 0.42.

**Table 4:** Correlation coefficient and *P* value of basic features defined from the incident and reflected waves, first inflection point, reconstructed PPG^a^, and original PPG.

Pulse type and feature		R^b^	*P* value
**Incident wave**			
	*INC* ^c^_peak-amp_	0.06	*P*=.104
	*INC*_area_	0.15	*P*<.001
	*INC*_peak-time_	0.23	*P*<.001
	*INC*_time_	0.18	*P*<.001
	*INC*_skew_	–0.16	*P*<.001
	*INC*_kurt_	–0.18	*P*<.001
**Reflected wave**			
	*REF* ^d^_peak-amp_	–0.42	*P*<.001
	*REF*_area_	–0.45	*P*<.001
	*REF*_peak-time_	0.10	*P*=.008
	*REF*_time_	0.02	*P*=.514
	*REF*_skew_	0.19	*P*<.001
	*REF*_kurt_	0.18	*P*<.001
**First inflection point**			
	*INF* ^e^_peak-amp_	–0.08	*P*=.022
	*INF*_peak-time_	0.18	*P*<.001
	*INF*_area_	–0.04	*P*=.275
**Reconstructed PPG**			
	*RPPG* ^f^_area_	–0.39	*P*<.001
	*RPPG*_skew_	0.40	*P*<.001
	*RPPG*_kurt_	0.04	*P*=.230
**Original PPG**			
	*SYS* ^g^_peak-amp_	0.02	*P*=.525
	*SYS*_peak-time_	0.27	*P*<.001
	*DIA* ^h^_peak-amp_	–0.39	*P*<.001
	*DIA*_peak-time_	0.24	*P*<.001
	*OPPG* ^i^_area_	0.06	*P*=.118
	*OPPG*_time_	0.08	*P*<.027
	*OPPG*_skew_	0.41	*P*<.001
	*OPPG*_kurt_	–0.07	*P*=.051

^a^PPG: photoplethysmogram.

^b^R: Pearson correlation coefficient.

^c^INC: incident wave.

^d^REF: reflected wave.

^e^INF: inflection point.

^f^RPPG: reconstructed photoplethysmogram.

^g^SYS: systolic.

^h^DIA: diastolic.

^i^OPPG: original photoplethysmogram.

**Table 5:** Correlation coefficient and *P* value of combined features created from the basic features.

Domain and feature		R^a^	*P* value
**Spatial**			
	*INC*^b^_peak-amp_/*INC*_area_	–0.18	*P*<.001
	*INC*_peak-amp_/*REF*^c^_peak-amp_	0.32	*P*<.001
	*INC*_peak-amp_/*REF*_area_	0.32	*P*<.001
	*INC*_peak-amp_/*RPPG*^d^_area_	0.28	*P*<.001
	*INC*_area_/*REF*_peak-amp_	0.38	*P*<.001
	*INC*_area_/*REF*_area_	0.37	*P*<.001
	*INC*_area_/*RPPG*_area_	0.34	*P*<.001
	*REF*_peak-amp_/*REF*_area_	0.19	*P*<.001
	*REF*_peak-amp_/*RPPG*_area_	–0.34	*P*<.001
	*REF*_area_/*RPPG*_area_	–0.34	*P*<.001
	*INC*_peak-amp_–*REF*_peak-amp_	0.31	*P*<.001
	*INC*_area_–*REF*_area_	0.34	*P*<.001
	*SYS*^e^_peak-amp_–*INC*_peak-amp_	–0.06	*P*=.104
	*SYS*_peak-amp_–*REF*_peak-amp_	0.42	*P*<.001
**Temporal**			
	*INC*_peak-time_/*INC*_time_	0.20	*P*<.001
	*INC*_peak-time_/*REF*_peak-time_	0.15	*P*<.001
	*INC*_peak-time_/*REF*_time_	0.23	*P*<.001
	*INC*_peak-time_/*OPPG*^f^_time_	0.22	*P*<.001
	*INC*_time_/*REF*_peak-time_	0.12	*P*<.001
	*INC*_time_/*REF*_time_	0.24	*P*<.001
	*INC*_time_/*OPPG*_time_	0.19	*P*<.001
	*REF*_peak-time_/*REF*_time_	0.16	*P*<.001
	*REF*_peak-time_/*OPPG*_time_	0.12	*P*<.001
	*REF*_time_/*OPPG*_time_	–0.19	*P*<.001
	*REF*_peak-time_–*INC*_peak-time_	0.03	*P*=.390
	*OPPG*_time_–*INC*_peak-time_	0.03	*P*=.369
	*OPPG*_time_–*REF*_peak-time_	0.03	*P*=.399
**Spatiotemporal**			
	*INC*_peak-amp_/*INC*_peak-time_	–0.28	*P*<.001
	*INC*_peak-amp_/*INC*_time_	–0.22	*P*<.001
	*INC*_peak-amp_/*REF*_peak-time_	–0.11	*P*=.002
	*INC*_peak-amp_/*REF*_time_	–0.02	*P*=.615
	*INC*_peak-amp_/*OPPG*_time_	–0.09	*P*=.015
	*INC*_area_/*INC*_peak-time_	–0.15	*P*<.001
	*INC*_area_/*INC*_time_	–0.11	*P*=.002
	*INC*_area_/*REF*_peak-time_	-0.05	*P*=.213
	*INC*_area_/*REF*_time_	0.02	*P*=.496
	*INC*_area_/*OPPG*_time_	–0.02	*P*=.542
	*REF*_peak-amp_/*INC*_peak-time_	–0.36	*P*<.001
	*REF*_peak-amp_/*INC*_time_	–0.32	*P*<.001
	*REF*_peak-amp_/*REF*_peak-time_	–0.22	*P*<.001
	*REF*_peak-amp_/*REF*_time_	–0.17	*P*<.001
	*REF*_peak-amp_/*OPPG*_time_	–0.22	*P*<.001
	*REF*_area_/*INC*_peak-time_	–0.40	*P*<.001
	*REF*_area_/*INC*_time_	–0.36	*P*<.001
	*REF*_area_/*REF*_peak-time_	–0.27	*P*<.001
	*REF*_area_/*REF*_time_	–0.25	*P*<.001
	*REF*_area_/*OPPG*_time_	–0.28	*P*<.001
	*RPPG*_area_/*INC*_peak-time_	–0.33	*P*<.001
	*RPPG*_area_/*INC*_time_	–0.28	*P*<.001
	*RPPG*_area_/*REF*_peak-time_	–0.17	*P*<.001
	*RPPG*_area_/*REF*_time_	–0.10	*P*=.005
	*RPPG*_area_/*OPPG*_time_	–0.16	*P*<.001

^a^R: Pearson’s correlation coefficient.

^b^INC: incident wave.

^c^REF: reflected wave.

^d^RPPG: reconstructed photoplethysmogram.

^e^SYS: systolic.

^f^OPPG: original photoplethysmogram.

### Statistical Results of Vascular Aging Assessment

**Figure 3:**
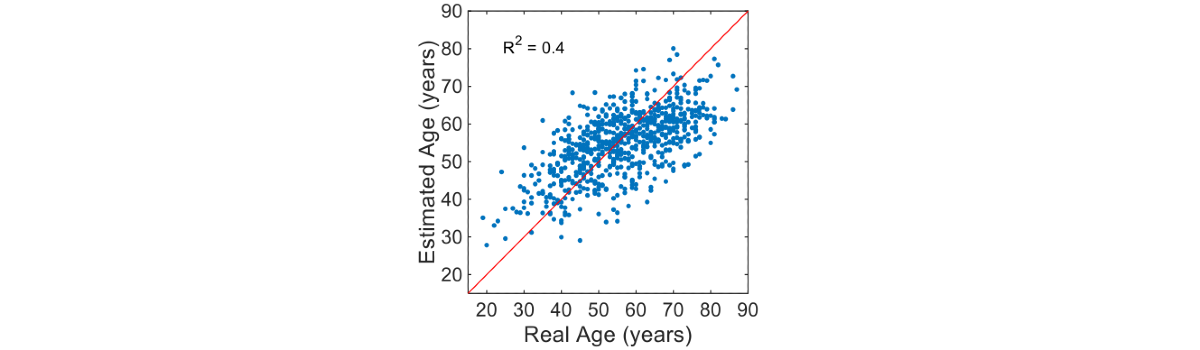
Scatter plot and coefficient of determination for the ANN-based regression model developed for the estimation of vascular aging in this study. ANN: artificial neural network.

**Figure 4:**
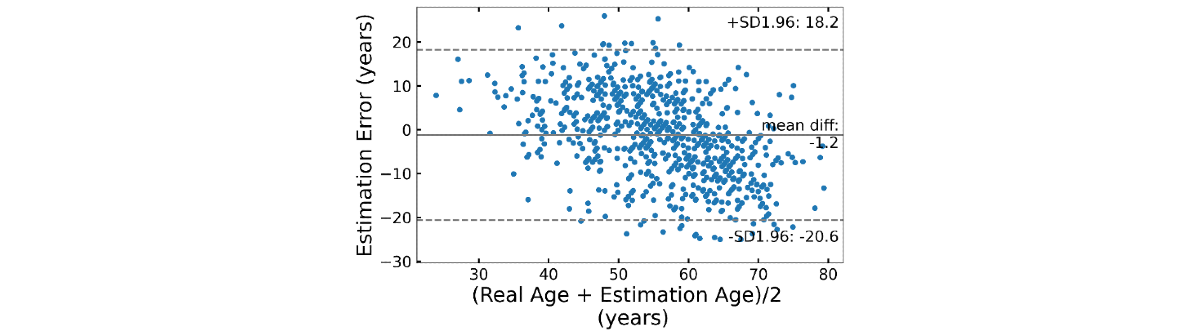
Bland-Altman plot for the ANN-based regression model developed for the estimation of vascular aging in this study. ANN: artificial neural network.

The RMSE for the age estimation of the ANN-based regression model developed in this study was 10.0 years. Figure 3 shows the scatter plot of the participant’s age estimated through the ANN-based regression model corresponding to the actual age and the coefficient of determination of the model. The estimated age and actual age of the ANN-based regression model showed a high correlation of 0.63 (*P*<.001), and the coefficient of determination of the model was 0.4. Figure 4 shows the Bland-Altman plot for the estimated age and the actual age through the ANN-based regression model developed in this study. The upper and lower limits of 95 % agreement were 18.2 and –20.6 years, respectively.

## Discussion

In this study, a highly correlated feature for assessing vascular aging was explored using features before and after decomposition of the incident and reflected waves of the PPG, and an ANN-based vascular aging estimation model was developed with the features derived. The ANN-based regression model showed the RMSE of 10.0 years in the age estimation. In comparing the correlation analysis before and after decomposition of the PPG incident and reflected waves, the feature defined after decomposition rather than before decomposition of the incident and reflected waves is useful for assessing vascular aging. In addition, in the comparison of all individual features, the feature defined from the reflected wave was confirmed as the best feature for assessing vascular aging. This reconfirms that changes in arterial stiffness due to vascular aging are reflected very well in the reflected wave characteristics of PPG, as Dawber et al [13] revealed. In the comparison of the correlation between the basic features defined from the PPG original waveform, incident wave, reflected wave, and reconstructed PPG waveform before and after PPG decomposition, *REF*_area_ and *REF*_peak-amp_ showed the highest correlation. These features are defined from the characteristic points of the reflected wave. In the results of our study, the time-related features of the reflected wave, such as *REF*_peak-time_ and *REF*_time_, showed a very weak correlation (*P*=.01) or no significant correlation (*P*=.51) with actual age, respectively. However, the amplitude-related feature of the reflected wave of *REF*_peak-amp_ and *REF*_area_ showed a weak correlation (|R|>0.3) with the actual age (R=–0.42 and R=–0.45 respectively). This result suggests that the amplitude-related feature of the reflected wave is more advantageous in estimating vascular aging than the time-related feature. The result in this study is different from the study of Millasseau et al [14,15], which found that SI, an index related to the temporal characteristic of the reflected wave, had a higher correlation with age than RI, an index related to the amplitude of the reflected wave. Also, in contrast to the results of Millasseau et al and Yousef et al [14-16], the results of this study showed that the amplitude of the reflected wave decreased with aging, and the RI decreased accordingly. Unlike the previous studies that used the PPG measured from the finger, it is thought that in this study, the use of the PPG measured from the nose had an effect. In a study by Hartmann et al [22], which observed changes in the main features of the PPG depending on the measurement location, it was reported that features such as RI could have a significant difference depending on the measurement location. Whether the change in the PPG waveform due to vascular aging has a specific pattern for each measurement location has yet to be clearly clarified; therefore, for clarification, additional research needs to be performed.

For the model development, hyperparameter optimization, such as number of hidden layers, number of nodes, dropout rate, learning rate, optimizer, early stopping patience, and input data scaler of the ANN-based regression model, was performed. In determining the hidden layer, as the number of hidden layers and the number of nodes in the hidden layer decreased, the age estimation error of the proposed model tended to decrease. In addition, as the dropout ratio of the hidden layer increased, the estimation error decreased. This means that the proposed model has sufficient expressive power to overfit the training data and that performance can be improved by suppressing overfitting [23,24]. For other training conditions used for model training, the model showed the highest performance when the optimizer was set to Adam, the learning rate was set to 0.001, and the early stopping patience was set to 50. In the case of the scaler that normalizes the input value, it was confirmed that the Robust scaler showed better performance. This is presumably because the input data contains many outliers. In comparing the performance of the model introduced in the previous study and the ANN-based regression model proposed in this study, the PPG features proposed by Millasseau et al and Yousef et al [14-16] weakly correlated with the actual age (R=–0.29 and R=–0.33 respectively). However, the ANN-based regression model proposed in this study strongly correlated with the actual age (R=0.63) and had better performance. In addition, the ANN-based regression model of this study had better performance than the previous studies in estimating the actual age of participants [18,19]. Similar to this study, the existing CNN model developed by Dall’Olio et al [18] with a single PPG input showed an estimation error of 12 years in RMSE, but the ANN-based regression model of this study showed a low estimation error of 10 years in RMSE. Moreover, our model has better estimation performance than the linear and ANN models using multiple inputs of PPG and ECG, showing estimation errors of 12 and 11 years, respectively [19] (see Table 6).

**Table 6:** Comparison of the proposed model to the models of previous studies in root mean squared error, correlation coefficient, and *P* value.

Reference and type of regression model		Input	RMSE^a^ (years)	R	*P* value
Proposed, ANN^b^		Features from raw PPG^c^ and incident and reflected wave separated from raw PPG	10	0.63	*P*<.001
Millasseau et al [14,15], linear		Feature from raw PPG	N/A^d^	–0.29	*P*<.001
Yousef et al [16], linear		Feature from raw PPG	N/A	–0.33	*P*<.001
Dall’Olio et al [18], CNN^e^		Raw PPG	12	N/A	N/A
Chiarelli et al [19]					
	Linear	Feature from raw PPG and ECG^f^	12	0.64	*P*<.001
	ANN	Feature from raw PPG and ECG	11	0.74	*P*<.001
	DCNN^g^	Raw PPG and ECG	7	0.92	*P*<.001

^a^RMSE: root mean squared error.

^b^ANN: artificial neural network.

^c^PPG: photoplethysmogram.

^d^N/A: not applicable.

^e^CNN: convolutional neural network.

^f^ECG: electrocardiogram.

^g^DCNN: deep convolutional neural network.

This study has some limitations. Most of the previous studies that performed vascular aging evaluation used finger PPG. However, in this study, vascular aging was evaluated based on nasal PPG. Therefore, it is difficult to generalize the results of this study to a vascular aging evaluation technique using PPG regardless of the measurement location. Therefore, it is necessary to analyze the aging-related waveform change characteristics of PPG obtained from various measuring sites through additional studies. In addition, the ANN-based regression model developed in this study for estimating vascular aging is a relatively simple machine learning model with one hidden layer. Therefore, in future studies, it is necessary to improve the vascular aging estimation performance by applying a more sophisticated machine learning technique with increased model complexity. Moreover, this study did not investigate various risk factors that can accelerate vascular disease, such as atherosclerosis; therefore, it is necessary to evaluate the model performance and examine the possibility of application according to various subject characteristics.

### Conclusion

In this study, we derived various features from the decomposed PPG waveforms before and after decomposition of the waveform into incident and reflected waves to explore features highly correlated with vascular aging, and it was confirmed that the reflected wave-related features had a strong correlation with participant’s age. In addition, the ANN-based regression model developed using the derived feature had 10 years of RMSE in estimating the participants’ actual age and showed the improved vascular aging estimation performance in comparison with the models introduced in previous studies. These results suggest that the developed technology can be applied to a wearable device and used to assess vascular health in real-life situations. However, this study was performed based on nasal PPG, not finger PPG, which is not frequently used in vascular aging evaluation studies. Since it is not clear whether the change in the PPG waveform due to vascular aging has a specific pattern for each measurement location, additional research needs to be performed for clarification.

## Abbreviations

AIx: augmentation index
ANN: artificial neural network
CNN: convolutional neural network
DCNN: deep convolutional neural network
DIA: diastolic
ECG: electrocardiogram
INC: incident wave
INF: inflection point
LOOCV: leave-one-out cross-validation
MAE: mean absolute error
OPPG: original photoplethysmogram
PPG: photoplethysmogram
REF: reflected wave
ReLU: rectified linear unit
RI: reflection index
RMSE: root mean squared error
RPPG: reconstructed photoplethysmogram
SI: stiffness index
SYS: systolic
